# Parapharyngeal and retropharyngeal infections in children: Kawasaki disease needs vigilance^☆^

**DOI:** 10.1016/j.bjorl.2024.101405

**Published:** 2024-02-22

**Authors:** Jia Liu, Shui-Hong Zhou

**Affiliations:** a Zhejiang University, College of Medicine, The First Affiliated Hospital, Department of Otolaryngology, Hangzhou, Zhejiang, China; b Children’s Hospital, Zhejiang University School of Medicine, National Clinical Research Center for Child Health, Department of Otolaryngology, Hangzhou, Zhejiang Province, China

**Keywords:** Kawasaki disease, Deep neck infections, Parapharyngeal infections, Retropharyngeal infections, Surgical timing

## Abstract

**Objective:**

Kawasaki Disease (KD) may mimic Parapharyngeal (PPI) and Retropharyngeal Infections (RPI), leading to misdiagnosis as Deep Neck Infections (DNIs). The treatment plans for the two diseases are different, and delayed treatment can lead to serious complications. Therefore, prompt diagnosis and management are necessary. This study was performed to evaluate the clinical features of KD mimicking DNIs and explore the treatment options.

**Methods:**

Children with cellulitis or abscess in parapharyngeal or retropharyngeal space in neck CT were included in this study. The medical records of enrolled children were retrospectively reviewed.

**Results:**

In total, 56 children were diagnosed with PPI or/and RPI. Twenty-two (39.3%) participants were eventually diagnosed with KD, and 34 (60.7%) were diagnosed with DNIs. Compared with the DNIs group, the KD group had a higher body temperature (*p* = 0.007), and higher levels of AST (*p* = 0.040), ALT (*p* = 0.027), and ESR (*p* = 0.030). Deep cervical cellulitis (*p* = 0.005) were more common in the KD group. However, deep neck abscess often occurred in the DNIs group (*p* = 0.002), with parapharyngeal abscess being the most common type of abscess (*p* = 0.004). The KD mimicking DNIs cases did not respond to antibiotic treatment, but symptoms significantly improved after the use of Immunoglobulin (IVIG) and aspirin.

**Conclusion:**

Children with KD may exhibit retropharyngeal or parapharyngeal inflammation in the early stages. KD should be considered a differential diagnosis for children with DNIs, high fever, and no response to antibiotic therapy. Surgery in KD mimicking deep neck abscess requires caution.

**Level of Evidence:**

I.

## Highlights

• Deep neck infections may manifest as an initial and atypical symptom of KD.
• Otolaryngologists should maintain awareness of the possible manifestations of KD.
• The operation should be cautious in cases that KD mimic deep neck abscesses.

## Introduction

Kawasaki Disease (KD), also known as skin-mucosal-lymph node syndrome, often occurs in infants and young children (younger than 5-years old).^1^ The incidence of USA patients with Kawasaki disease was 1.9 per 10,000 children,^2^ and Asians are 2.5 times more than USA.^3^ Due to the lack of specific diagnostic tests, the diagnosis of KD is mainly based on its main clinical symptoms.^3^ There are at least 4 of 5 principal clinical criteria, including non-purulent bulbar conjunctivitis, changes in the lips or oral cavity, polymorphous exanthema, erythema with later desquamation of the extremities, and at least one cervical lymph node > 1.5 cm in size, with prolonged (≥5-days) high fever. However, patients could have “incomplete KD” in the presence of 2 or 3 principal symptoms accompanied by high fever for at least five days.^4^ This is often challenging, as only 40% of patients with KD present with conclusive clinical criteria, and others present with incomplete or atypical symptoms.^5^ Some case reports indicated that KD can initially manifest as parapharyngeal^6^, ^7^, ^8^ or/and retropharyngeal^4^, ^9^, ^10^, ^11^ infections, leading to misdiagnosis as Deep Neck Infections (DNIs).^12^, ^13^ DNIs may be the initial presenting symptom before conclusive clinical criteria of KD appeared.^14^ Rim Kasem Ali Sliman et al. considered DNIs as an atypical presentation of KD.^11^

Deep Neck infections (DNIs) include all infections in the potential spaces and fascial planes of the neck beneath the deep layer of the cervical fascia, with Parapharyngeal Space Infections (PPI) and Retropharyngeal Space Infections (RPI) being the most common.^15^, ^16^ A national study carried out in the USA has shown that the incidence of pediatric DNIs was 1.07–1.37 cases per 10,000.^17^ Due to the specificity of the anatomical structure of the parapharyngeal and retropharyngeal spaces, infections may lead to serious respiratory and vascular problems, leading to rapid progression and life-threatening complications.^18^

Although KD is a self-limited vasculitis, delayed diagnosis and treatment may lead to life-threatening complications, such as Coronary Artery Aneurysms (CAAs) or myocardial infarction, and KD is the main cause of acquired heart disease in children.^19^ The incidence of CAA decreases to <5% after timely treatment with high-dose intravenous immunoglobulin (2 g/kg/day), which is most effective within the first ten days of symptoms.^20^ Awareness of unusual clinical manifestations is important, as it may raise the index of suspicion and expedite treatment.^10^, ^20^, ^21^

The treatment methods of KD and DNIs are quite different. Prompt diagnosis and management are necessary. This study was performed to evaluate the clinical features of KD mimicking DNIs. At the same time, we explored the treatment options. Through the summarized of clinical characteristics and discussion of treatment, the otolaryngologist should be aware of the possible symptoms of KD and should include the disease in the differential diagnosis of deep neck infections.

## Methods

### Patients

From January 2020 to December 2022, 76 children with parapharyngeal or retropharyngeal cellulitis or abscess in neck CT were initially enrolled in this study. After excluding cases of trauma, cervical structural anomalies, and clear confounding factors, 56 cases were included in this study. This study was approved by the Ethics Committee, and informed consent was waived. The inclusion evaluation team consisted of radiologists, otolaryngologists, and cardiologists.

### Data collection

**Figure 1:**
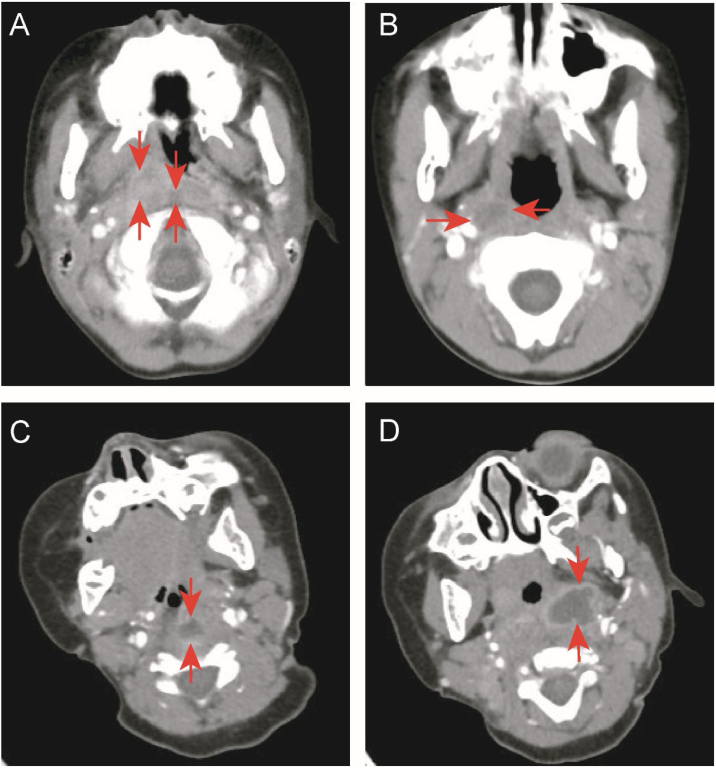
Enhanced neck computed tomography. (A) Retropharyngeal and right parapharyngeal cellulitis (arrowheads). (B) Right parapharyngeal cellulitis (arrowheads). (C) Retropharyngeal abscess with rim enhancement (arrowheads). (D) Left parapharyngeal abscess with rim enhancement (arrowheads).

This study retrospectively analyzed the medical records of enrolled children. We collected demographic data, including gender and age, and clinical data, including the final diagnosis, fever duration before and after admission, accompanying symptoms, head and neck examination results, treatment methods, and outcomes. White Blood Cell Count (WBC), C-Reactive Protein (CRP), absolute neutrophil count, absolute monocyte count, hemoglobin level, platelet count, Erythrocyte Sedimentation Rate (ESR), pre-calcitonin, Lactate Dehydrogenase (LDH), Aspartic Acid Transaminase (AST), and Alanine Transaminase (ALT) levels, and other data were collected. The location (parapharyngeal or retropharyngeal space) and type (cellulitis or abscess) of deep neck inflammation were determined based on enhanced neck CT. The basis for determining cellulitis or abscess came from Kyungmin Roh et al.^22^: Cellulitis was defined as the presence of unreinforced low-density lesions in the parapharyngeal or retropharyngeal space, while abscess was defined as the presence of low-density lesions with completely enhanced edges (Fig. 1).

This study analyzed the cases of KD mimicking deep neck abscess in children who underwent incision and drainage surgery. We collected the basic information, disease course, treatment process, and prognosis of the patients to analyze the timing and effectiveness of surgery in KD mimicking deep neck abscesses.

### Statistical analysis

The collected children were divided into DNIs and KD groups based on the final diagnosis before being discharged. Demographic, clinical, and laboratory data were compared between the two groups. Categorical and continuous variables were compared using Fisher’s exact and Independent sample *t*-tests, respectively. The SPSS version 22.0 (IBM Corp., USA) was used for statistical analyses, and statistical significance was defined as *p* < 0.05.

## Results

### Demographic and clinical data

We initially collected 76 children with cervical CT findings in favor of cellulitis and/or abscess in the parapharyngeal or retropharyngeal space. Two cases with incomplete data and all cases with clear confounding factors, including 5 children with immunosuppression due to hematologic diseases and cancer, 3 cases with neck trauma, 3 cases with pyriform sinus fistula, 2 cases with immunodeficiency, 2 cases with Kikuchi disease (pathologically confirmed), 1 case with connective tissue disease, 1 case with type I diabetes, and 1 case with spinal muscular atrophy, were excluded, and 56 cases remained.

The median age of the collected children was 5-years and 6-months (range 9-months to 10-years), and 30 (53.6%) children had male gender. The most common clinical symptoms were fever 55 (98.21%), neck pain 45 (80.4%), and limited neck motion 36 (64.3%). Other symptoms, such as sore throat 16 (28.6%) or cough 10 (17.9%) occurred occasionally. Trismus 7 (12.5%), anhelation or snore 7 (12.5%), dysphagia 6 (10.7%), headache 6 (10.7%), sputum discharge 6 (10.7%), and rhinorrhea 3 (5.4%) were less. All patients had cervical lymphadenopathy 56 (100%); the vast majority of patients had Pharyngeal congestion 51 (91.1%); and nearly half of the physical examinations showed asymmetrical protrusions and pharyngeal tonsil deviation 26 (46.4%) (Table 1).

**Table 1:** Clinical features of deep neck infection in children. Values are presented as number (%) or mean ± standard deviation.

Factor	n (%)	Means
Gender		
Male	30 (53.6%)	
Female	26 (46.4%)	
Age (month)		68.04 ± 29.89
Symptom		
Fever (°C)	55 (98.21%)	39.27 ± 0.73
Fever duration (day)		9 ± 4.14
Cough	10 (17.9%)	
Sputum	6 (10.7%)	
Rhinorrhea	3 (5.4%)	
Sore throat	16 (28.6%)	
Neck pain	45 (80.4%)	
Limited neck motion	36 (64.3%)	
Trismus	7 (12.5%)	
Dysphagia	6 (10.7%)	
Anhelation or snore	7 (12.5%)	
Headache	6 (10.7%)	
Sign		
Pharyngeal congestion	51 (91.1%)	
Tonsillar deviation	26 (46.4%)	
Cervical lymphadenopathy	56 (100%)	
Computed tomography finding		
Cellulitis		
Parapharyngeal cellulitis	18 (32.1%)	
Retropharyngeal cellulitis	15 (26.8%)	
Abscess		
Parapharyngeal abscess	26 (46.4%)	
Retropharyngeal abscess	13 (23.2%)	
Combination of antibiotics	31 (55.4%)	
Undergo surgery	7 (12.5%)	

Deep neck abscess and cellulitis were identified in neck CT of 31 (55.4%) and 26 (46.4%) children, respectively. One child (1.9%) had both deep neck abscess and cellulitis. Eight (25.8%) of the 31 children with deep neck abscesses had concurrent parapharyngeal and retropharyngeal abscesses. Seven (26.9%) of the 26 children with deep neck cellulitis had concurrent parapharyngeal cellulitis and retropharyngeal cellulitis. Deep neck abscess was defined as parapharyngeal abscesses in 26 (83.9%) cases and retropharyngeal abscesses in 13 (41.9%) cases. There were 18 (69.2%) cases of parapharyngeal cellulitis and 15 (57.7%) cases of retropharyngeal cellulitis among deep neck cellulitis (Table 2).

**Table 2:** Type of deep neck inflammation.

Factor	Number
Parapharyngeal abscess	17
Retropharyngeal abscess	5
Parapharyngeal cellulitis	11
Retropharyngeal cellulitis	7
Parapharyngeal abscess + retropharyngeal abscess	8
Parapharyngeal cellulitis + retropharyngeal cellulitis	7
Parapharyngeal abscess + retropharyngeal cellulitis	1
Total	56

### Comparison between DNIs and KD groups

Among 56 collected children, the final diagnosis was KD in 22 cases (39.3%) and DNIs in 34 cases (60.7%). Twenty-two children with KD and initial symptoms of deep neck infection were diagnosed after observing characteristic clinical manifestations in the later stages. Among them, 19 cases had 2 or 3 clinical features of KD, which appeared within a median of 6-days after fever (range 4–15). However, three patients did not show characteristic clinical manifestations of KD, and physicians diagnosed KD based on the duration of fever and coronary artery abnormalities on echocardiography. The median duration of fever on admission for children diagnosed with KD was 5-days (range 2–13 days), and the median duration of fever after their admission was 4-days (range 1–11 days).

There was no significant difference in gender and age between the DNIs and KD groups. There was a significant difference in the highest body temperature between the two groups (*p* = 0.007), and the KD group had a higher body temperature. Regarding the clinical symptoms, dysphagia is rare in KD group (*p* = 0.041) compared to DNIs group.

Laboratory results upon admission revealed significant elevations in WBC, CRP, and absolute neutrophil count in both groups, with no significant distinction between them (*p* > 0.05). Furthermore, ESR levels were significantly elevated in both groups, with a notably higher level in the KD group when compared to the DNIs group (*p* = 0.030). The KD group exhibited higher levels of AST (*p* = 0.040) and ALT (*p* = 0.027) compared to the DNIs group. In addition, the KD group displayed a higher incidence of deep cervical cellulitis (*p* = 0.005) on neck CT scans, whereas deep neck abscesses were more common in the DNIs group (*p* = 0.002), with parapharyngeal abscesses being the most frequently observed (*p* = 0.004) (Table 3).

**Table 3:** Comparison between children in the DNI and KD groups.

Factor	DNI group (n = 34)	KD group (n = 22)	*p*-value
Gender			0.071
Age (month)	68.71 ± 30.16	67.00 ± 30.16	0.837
Symptom			
Fever (°C)	38.98 ± 0.86	39.58 ± 0.60	0.007
Fever duration (day)	8.71 ± 4.79	10.14 ± 3.30	0.226
Cough	7 (20.6%)	3 (13.6%)	0.387
Sputum	5 (14.7%)	1 (4.5%)	0.230
Rhinorrhea	2 (5.9%)	1 (4.5%)	0.661
Sore throat	12 (35.3%)	4 (18.2%)	0.139
Neck pain	27 (79.4%)	18 (81.8%)	0.555
Limited neck motion	22 (64.7%)	14 (63.6%)	0.578
Trismus	5 (14.7%)	2 (9.1%)	0.428
Dysphagia	6 (17.6%)	0 (0%)	0.041
Anhelation or snore	6 (17.6%)	1 (4.5%)	0.151
Headache	4 (11.8%)	2 (9.1%)	0.560
Sign			
Pharyngeal congestion	29 (85.3%)	22 (100%)	0.073
Tonsillar deviation	16 (47.1%)	10 (45.5%)	0.563
Blood test			
White blood cell count (×10^9^/L) (Reference values: 4.00–12.00)	19.98 ± 7.46	18.05 ± 7.17	0.341
C-reactive protein (mg/L) (Reference values: 0.00–8.00)	72.08 ± 49.76	101.22 ± 66.09	0.066
Absolute neutrophil count (×10^9^/L) (Reference values: 1.50–7.80)	13.75 ± 7.54	13.72 ± 5.08	0.988
Absolute monocyte count (×10^9^/L) (Reference values: 0.10–1.50)	1.21 ± 0.49	1.05 ± 0.46	0.232
Hemoglobin (g/L) (Reference values: 110–155)	118.21 ± 13.48	113.09 ± 13.55	0.172
Platelet count (×10^9^/L) (Reference values: 100–400)	394.94 ± 108.10	333.36 ± 166.16	0.098
Aspartate transaminase (IU/L) (Reference values: 14–44)	28.59 ± 11.62	52.50 ± 50.70	0.04
Alanine transaminase (IU/L) (Reference values: 7–30)	16.00 ± 16.91	34.50 ± 34.60	0.027
Lactate dehydrogenase (IU/L) (Reference values: 110–295)	278.79 ± 135.29	329.23 ± 182.84	0.240
Erythrocyte sedimentation rate (mm/h) (Reference values: 0–20)	67.04 ± 29.52	85.14 ± 30.04	0.030
Procalcitonin(ng/mL) (Reference values: 0‒0.460)	0.36 ± 0.78	0.60 ± 0.49	0.198
Computed tomography finding			
Cellulitis	15 (44.1%)	18 (81.8%)	0.005
Parapharyngeal cellulitis	8 (23.5%)	10 (45.5%)	0.078
Retropharyngeal cellulitis	7 (20.6%)	8 (36.4%)	0.160
Abscess	29 (85.3%)	10 (45.5%)	0.002
Parapharyngeal abscess	21 (61.8%)	5 (22.7%)	0.004
Retropharyngeal abscess	8 (23.5%)	5 (22.7%)	0.605
Combination of antibiotics	19 (55.9%)	12 (54.5%)	0.569
Undergo surgery	5 (14.7%)	2 (9.1%)	0.428

DNI, Deep Neck Infection; KD, Kawasaki Disease. Values are presented as number (%) or mean ± standard deviation. Categorical and continuous variables were compared using Fisher’s exact and independent sample *t*-tests, respectively; *p* < 0.05 was considered statistically significant.

### Treatment and outcomes of the DNIs group

Among the 34 cases of DNIs, 29 cases (85.3%) were cured after antibiotic therapy, of which 19 cases (55.9%) were treated with a combination of the two drugs. The median duration of fever after treatment was 2.5 days (range 0–14 days).

In the DNIs group, 5 patients (14.7%) underwent surgical intervention. Four patients underwent oropharyngeal incision and drainage, and one patient had a deep abscess and underwent ultrasound-guided drainage. The 5 children who underwent surgical treatment were all diagnosed with large abscess, manifesting with respiratory obstruction and snoring. Therefore, incision and drainage were considered to protect respiratory and airway.

### Treatment and outcomes of the KD group

Twenty-two children finally diagnosed as KD were treated with intravenous immunoglobulin (IVIG, 2 g/kg) and aspirin (80 mg/kg). The median duration of fever after treatment was 3-days (range 1–8 days). Among them, there were 5 patients whose body temperature did not improve within 3 days after treatment. Among them, two patients improved on the 4th day, and one patient improved on the 5th day but experienced Kawasaki shock syndrome and liver injury. One patient improved on the 7th day and was defined as unresponsive Kawasaki. One patient improved on the 8th day and was defined as unresponsive Kawasaki, experiencing knee arthritis in the later stage. Children with KD who have a high body temperature for more than 3 days after using IVIG and aspirin have a relatively poor prognosis. In addition, 12 (54.5%) children in the KD group received a combination of two antibiotics, and ten cases (45.5%) received one antibiotic for suspected deep neck infections before being diagnosed with KD. However, there was no significant improvement in clinical symptoms, such as fever, after antibiotic use.

**Figure 2:**
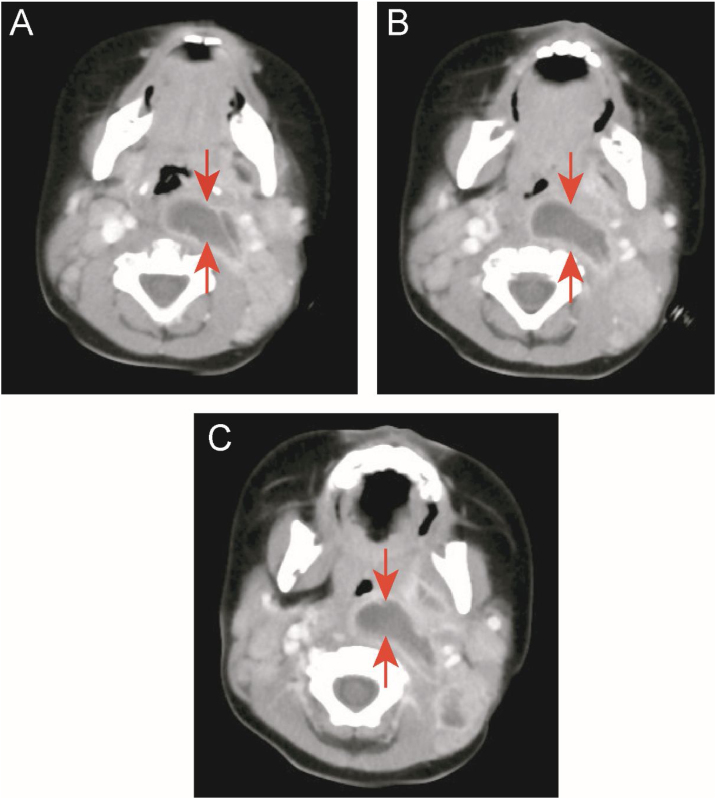
Case 1: a patient with KD and deep neck abscess who underwent incision and drainage surgery. (A, B, and C) Enhanced neck computed tomography showing a neck abscess.

Two children (9.1%) in the KD group underwent oropharyngeal incision and drainage surgery due to respiratory obstruction and snoring. One of the cases was a 10-month-old boy admitted to the hospital for 13 days due to high fever (up to 40 °C), left neck pain, and upper respiratory obstruction. Early use of antibiotics did not resolve the fever. On the 15th day of fever, KD was diagnosed by echocardiography. His body temperature improved 4 days after receiving IVIG and aspirin, but the abscess still did not shrink. After one week of close observation, CT still showed a large abscess in the neck, and snoring was still severe. After incision and drainage, breathing became smooth. However, there was no bacterial growth in the drainage culture (Fig. 2 A–C).

**Figure 3:**
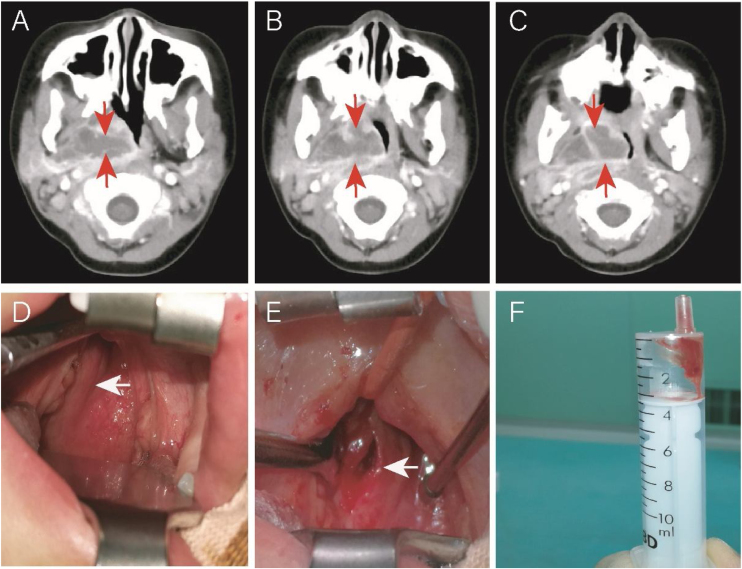
Case 2: a patient with KD and deep neck abscess who underwent incision and drainage. (A, B, and C) Enhanced neck computed tomography showing a neck abscess. (D) Right parapharyngeal and retropharyngeal eminence (arrowhead). (E) After incision and drainage (arrowhead). (F) One milliliter of serous fluid was drained without bacterial growth in cultures.

Another case was a 4-year-old (50-months old) female without comorbidities admitted to the hospital for 5-days due to fever (39.5–40 °C), right neck pain, upper respiratory obstruction, and snoring. On the 10th day of fever, echocardiography showed dilation of the left main coronary artery, confirming KD. IVIG and aspirin were immediately given. On the same day, an neck CT showed a parapharyngeal abscess. Due to the significant compression of the pediatric airway by the abscess, the patient was transferred to the Otolaryngology Department for surgery (Fig. 3 A–C). Her fever subsided 24 h after IVIG infusion (37.6 °C), and the upper respiratory tract obstruction was alleviated. Otolaryngologists and anesthesiologists were not sure if the surgery was necessary. Due to the possibility of upper airway obstruction and abscess rupture, surgery was performed on the 13th day of fever (36 h after initiating treatment with IVIG and aspirin). Surprisingly, during the surgery, it was found that the right pharyngeal wall and tonsil were significantly smaller than before using IVIG. The abscess (approximately 1 cm deep) was discharged through oral incision and drainage. However, only 1 mL of viscous bloody fluid was discharged (Fig. 3 D–F), resulting in significant intraoperative bleeding (approximately 7 mL). We expanded the incision by approximately 1.0 cm. No bacterial growth was found in the pus culture. Postoperative upper respiratory tract obstruction was alleviated. Due to parapharyngeal surgery, the patient received a liquid diet through a nasogastric tube for 3 days.

## Discussion

Diagnosing cases with typical KD clinical manifestations is relatively straightforward, but the prevalence of “incomplete KD” has been on the rise in recent years.^3^, ^11^ Some researchers have proposed that a DNI-like presentation is an atypical symptom of KD.^5^ While DNI-like presentations of KD seem to be rare (occurring in less than 5% of all patients with head and neck manifestations of KD),^11^ the actual incidence of parapharyngeal or retropharyngeal pathology remains unknown due to the lack of routine neck imaging for KD. Consequently, the likely prevalence of unreported cases also remains uncertain. Epidemiological studies show that the prevalence of NDIs is very low.^17^ We reviewed 3-years and only obtained 56 matching cases. More than a third of children initially diagnosed with DNIs based on neck CT scans were ultimately diagnosed with KD. There are several probable reasons. 1) The information we gathered was on Chinese children. The incidence of Asians patients with Kawasaki disease are 2.5 times more than USA.^3^ Similar to a study in South Korea^14^ (another Asian country), 11 of 47 (23.4%) children initially diagnosed with DNIs were ultimately diagnosed with KD. 2) Following the practical administration of broad-spectrum antibiotics, the prevalence of DNIs in children reduced dramatically.^4^ This may induce the proportion of KD simulated DNIs relatively higher. 3) In the NDIs that were eventually included, we carefully excluded individuals with other disorders that potentially affect the development of DNIs. Such include inadequate immunity, trauma, and congenital structural defects. Rim Kasem Ali Sliman et al.,^11^ in their literature search using keywords such as “Kawasaki disease”, “pediatrics”, and “retropharyngeal abscess”, found fifteen articles, with sixteen case reports describing pediatric patients presenting with retropharyngeal abscesses who were later diagnosed with Kawasaki disease. This suggests that, in some cases, DNI-like manifestations may serve as an initial and atypical symptom before clear KD clinical criteria emerge.

When the diagnosis of KD based on typical symptoms is inconclusive, certain laboratory indicators can provide valuable reference points^3^, ^4^: WBC ≥ 15 × 10^9^/L, predominantly with mature and immature neutrophils; CRP levels > 30 mg/L, with or without ESR exceeding 40 mm/h; decreased hemoglobin; elevated aminotransferase levels; PLT > 450 × 10^9^/L 7-days after the onset. We examined the differences in laboratory indicators between the KD group and the DNIs group and explored the significance of these indicators in differential diagnosis. In our study, WBC count, CRP levels, absolute neutrophil count, and ESR were significantly elevated in both groups. No signs of anemia or platelet abnormalities were detected. Transaminase levels in the KD group were higher compared to the DNIs group. After reviewing relevant literature, we noted that some studies suggested that CRP, AST, and ALT levels were higher in KD children who initially presented with fever and neck lymphatic swelling as their primary manifestations.^23^, ^24^, ^25^, ^26^, ^27^ These findings indicate that elevated aminotransferase levels may serve as a differentiating diagnostic indicator between the two groups. On the other hand, the most common clinical signs and symptoms were similar in both the KD and DNIs groups and were insufficient for distinguishing between the two.

Consistent with previous studies, we found that KD mainly causes deep cervical cellulitis rather than abscess.^13^, ^23^, ^25^ The pathophysiological mechanism of parapharyngeal and retropharyngeal inflammation and edema in KD is still unclear. However, systemic vasculitis and increased microvascular permeability are considered to be the main mechanisms.^28^ Previous studies have found that the alterations caused by KD in MRI are most common due to vascular edema.^29^ Ueda et al.^30^ believed that the posterior wall of the pharynx consists of loose connective tissue and is prone to edema. The swollen lymph nodes impair lymphatic circulation, and lymph accumulates in the posterior wall of the pharynx, forming a low-density edematous area. The imaging appearance is similar to that of an abscess in the posterior wall of the pharynx.^22^ Therefore, almost all KD simulated DNIs medical records report that antibiotic treatment is ineffective^4^, ^6^, ^7^, ^9^^,^^10^, ^14^ and will be absorbed after treatment with IVIG and aspirin.^10^, ^13^

Cai et al.^6^ believe that KD mimicking deep neck abscess is not an indication for surgery. These patients should not be promptly scheduled for surgery. For patients with upper airway compression caused by abscesses, short-term intubation should be performed until local inflammation and swelling subside.^4^, ^6^ In our study, KD mimicking deep neck abscess improved significantly after IVIG and aspirin treatment in most patients, and only two patients underwent surgery. The reason for surgery was difficulty breathing. Both cases were misdiagnosed as DNIs in the early stage, and there were delays in the treatment of KD. Surgery can be considered for KD children with a large abscess cavity, significant compression, and no significant reduction after treatment. However, more data are needed to support these findings.

## Conclusion

Retropharyngeal and/or parapharyngeal inflammation may manifest as an initial and atypical symptom preceding the emergence of clear clinical criteria for KD in children. It is crucial to consider KD as a potential differential diagnosis when encountering children with DNIs, particularly those with cellulitis, characterized by high fever and unresponsiveness to antibiotic therapy. Elevated ESR and transaminase levels may also serve as warning indicators. Otolaryngologists should maintain awareness of the possible manifestations of KD and should include this condition in their list of differentials for DNIs. Caution is advised when surgical intervention is contemplated in cases that mimic deep neck abscesses but are ultimately attributed to KD.

## Funding

This work was supported by the Zhejiang Provincial Medical and Health Technology Plan Project (Grant number 2022KY863).

## Conflicts of interest

The authors have no other funding, financial relationships, or conflicts of interest to disclose.
